# Phase Separation: Orchestrating Biological Adaptations to Environmental Fluctuations

**DOI:** 10.3390/ijms26104614

**Published:** 2025-05-12

**Authors:** Wenxiu Wang, Fangbing Han, Zhi Qi, Chunxia Yan, Bodan Su, Jin Wang

**Affiliations:** 1 National Key Laboratory of Agricultural Microbiology, Biotechnology Research Institute, Chinese Academy of Agricultural Sciences, Beijing 100081, China; 2 Key Laboratory of Forage and Endemic Crop Biology, Ministry of Education, School of Life Science, Inner Mongolia University, Hohhot 010021, China; 3 College of Agriculture, Henan University, Kaifeng 475004, China

**Keywords:** phase separation, biomolecular condensates, weak interactions, stress resistance, environmental adaptability

## Abstract

Organisms have evolved various protective mechanisms to survive in complex and dynamic environments. Phase separation is a ubiquitous mechanism in plants, animals, and microorganisms. It facilitates the aggregation of biomolecules through weak interactions, forming membrane-less organelles that help organisms respond effectively to stress signals. These biomolecular condensates include DNA, RNA, and proteins. Proteins are categorized into scaffold and client proteins, whose coordinated actions ensure the compartmentalization of cellular signals, thereby regulating various biological processes. Studies indicate that, in response to stress, phase separation modulates gene expression, signal transduction, cytoskeleton dynamics, and protein homeostasis, ensuring the precise spatiotemporal control of cellular functions. These insights underscore the crucial role of phase separation in maintaining cellular integrity and adaptability.

## 1. Introduction

Organisms constantly face environmental fluctuations, such as temperature changes, osmotic stress, and oxidative damage. To survive and reproduce under these conditions, cells use mechanisms that ensure precise timing and efficiency of protein interactions and biochemical reactions. Membrane-bound organelles provide a specific microenvironment for protein interactions and biochemical reactions, and their membrane structures separate different processes to avoid interference [1]. This compartmentalization ensures that reactions occur efficiently and without cross-reactions, maintaining cellular organization and function [2]. In addition to membrane-bound organelles, cells utilize phase separation to form liquid-like, membrane-less condensates [3]. These condensates concentrate specific proteins and nucleic acid molecules, thereby promoting or inhibiting specific biochemical reactions [4]. Moreover, the dynamic nature of membrane-less condensates allows frequent molecular exchange with the surrounding environment. This dynamic equilibrium enables cells to respond rapidly to environmental changes.

Phase separation, a regulatory mechanism found in plants, animals, and microorganisms, is crucial for enhancing the efficiency and accuracy of signal transduction pathways. It enables cells to adapt effectively to environmental changes by organizing the signaling molecules into dynamic condensates. In cellular biology, phase separation involves the demixing of biomolecules like proteins and nucleic acids to form distinct structures [3,5]. This spatial and temporal organization is vital for signal transduction, thus ensuring timely and accurate information relay for appropriate cellular responses. By concentrating signaling molecules, phase separation enhances reaction rates and reduces erroneous interactions, thereby supporting cellular adaptation and survival.

**Figure 1:**
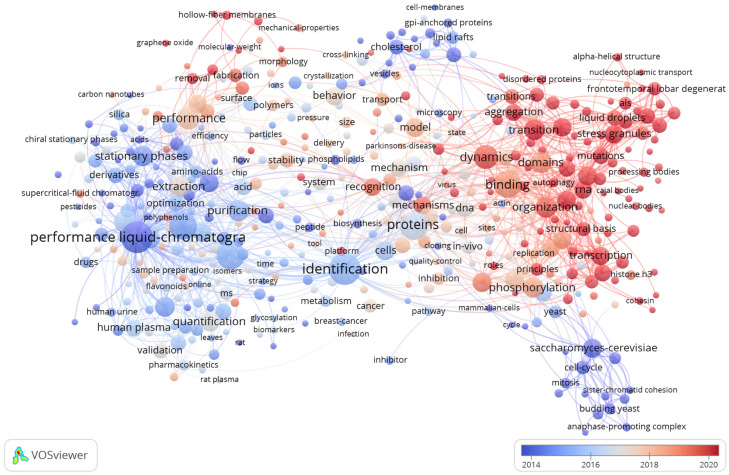
Temporal evolution of research keywords in the phase separation field. This figure presents a comprehensive visualization of the research keywords in the phase separation domain derived from extensive big data analysis. Different colors are used to distinguish keywords across various years from 2010 to 2024, facilitating an intuitive understanding of the temporal shifts in the research focus. The font size of each keyword is proportional to the volume of research associated with it; larger fonts indicate more extensive research and thus represent hotspots in the field.

In recent years, research on the mechanisms of phase separation has deepened (Figure 1). The period from 2010 to 2017 marked a foundational phase in phase separation research, primarily focusing on establishing phase separation as a biologically relevant phenomenon and identifying key proteins capable of forming membrane-less condensates. During this period, studies emphasized biophysical characterization using techniques such as in vitro reconstitution assays and fluorescence recovery after photobleaching (FRAP), which confirmed the dynamic nature of the condensates. From 2018 to 2024, the field transitioned into a mechanistic exploration phase, with research pivoting toward resolving the molecular determinants of phase separation and its functional consequences in stress adaptation. Key advances include stress granules, dynamics, domain-specific drivers, and structural insights. This shift underscores the progression from phenomenological observations to mechanistic dissection. The refined focus on sequence-encoded phase separation rules and condensate functional plasticity has established phase separation as a central paradigm in cellular organization and adaptive responses.

In plants, phase separation has been observed in various processes, including stress responses and developmental signaling. For instance, during periods of drought or high salinity, plants must quickly adjust their physiological processes to survive. Phase-separated condensates can help organize the stress response machinery, allowing plants to efficiently manage the complex signaling networks involved in adaptive responses. FLOE1 can sense changes in environmental moisture and regulate seed germination through phase separation, which helps plants adapt to dry terrestrial environments [6].

In animals, phase separation is crucial for processes such as embryonic development and neuronal signaling. It helps to organize the intricate networks of proteins and other molecules required for cell differentiation and communication. In the nervous system, for example, phase-separated structures can facilitate the rapid transmission of signals across synapses, ensuring swift and accurate neural responses to stimuli. Dysregulation of phase separation is a key factor in the pathogenesis of neurodegenerative diseases. In amyotrophic lateral sclerosis (ALS), the FUS protein typically aids DNA repair through phase separation [7,8]. However, mutations can lead to abnormal aggregation in the cytoplasm, forming pathological inclusions that disrupt normal cell functions and trigger ALS. Similarly, TDP-43 protein abnormalities contribute to neuronal damage in ALS and frontotemporal dementia (FTD) [9,10]. These disruptions underscore the importance of proper phase separation in maintaining neuronal health.

Microorganisms also utilize phase separation to adapt to environmental changes. Bacteria, for instance, can form phase-separated compartments to regulate metabolic pathways or respond to nutrient availability. This ability to dynamically reorganize cellular components allows microorganisms to thrive under diverse and often challenging conditions [11,12,13,14]. In response to environmental changes, fungal cells form membrane-less organelles through phase separation, which play crucial roles in regulating metabolism and cellular adaptability. Under low pH conditions, proteasomes and free ubiquitin in yeast cells aggregate into droplets, forming proteasome storage granules (PSGs), which help cells survive in extreme conditions. These granules also confer adaptability to cells during the aging process. Although the role of phase separation in microbial biological processes has gradually attracted attention, related research remains relatively scarce and requires further in-depth exploration.

Hence, understanding the role of phase separation in how organisms adapt to environmental fluctuations is crucial for unveiling the complexity of life systems and developing new strategies to combat diseases and improve human health.

## 2. The Basic Principle of Phase Separation

### 2.1. The Concept and Properties of Phase Separation

Phase separation, a concept in physical chemistry, describes dynamic processes in which changes in external conditions and composition characteristics cause a homogeneous system to spontaneously separate into distinct phases with different compositions and physical properties. In 2009, Hyman et al. discovered that P granules in Caenorhabditis elegans embryos, composed of RNA and proteins, form droplets via phase separation [15]. This introduced the concept of phase separation in biology, where biomolecules like proteins, nucleic acids, and lipids form membrane-less organelles or biomolecular condensates through multivalent interactions in a homogeneous environment [16]. Biomacromolecules undergo phase separation to create dynamic condensates that are essential for cellular organization. These structures enable the compartmentalization of biochemical reactions, influencing processes such as gene expression and stress responses [3,17,18].

**Figure 2:**
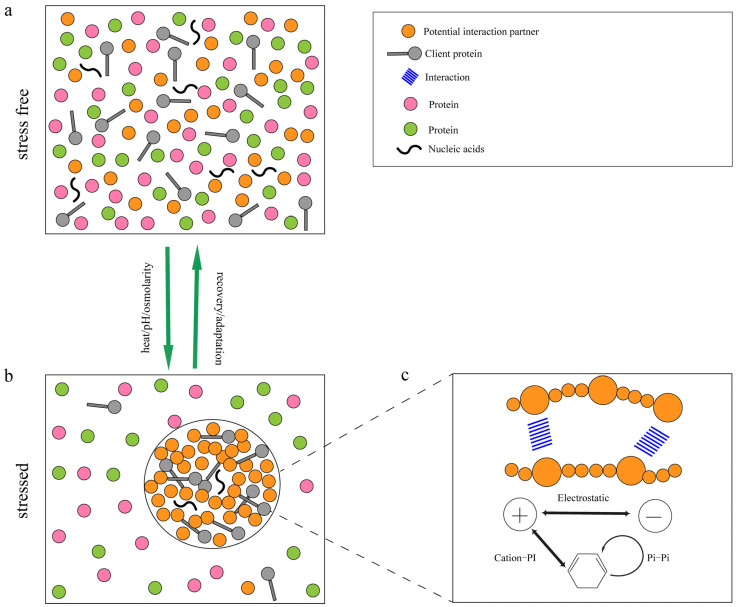
Protein interactions and multivalent interaction-driven phase separation under stress conditions. In the absence of stress, proteins and other molecules are uniformly distributed within the cell (**a**). Upon exposure to stress, proteins with the potential for phase separation can recruit ligand proteins to form condensates (**b**). The formation of these condensates is driven by protein–protein interactions and cation–Pi or Pi–Pi stacking interactions (**c**). When the stress is removed, the condensates disassemble, and proteins and other molecules return to a uniform distribution (**a**). This process illustrates how cells dynamically respond to and adapt to environmental stress through phase separation mechanisms. π–π stacking: illustrated as parallel/displaced aromatic ring interactions (e.g., phenylalanine or tyrosine side chains) that stabilize hydrophobic clustering within condensates. Cation-π interactions: depicted as electrostatic attractions between positively charged residues (e.g., lysine/arginine) and electron-rich aromatic rings, emphasizing their role in orienting biomolecular assemblies.

Cellular condensates dynamically assemble and disassemble in response to the physiological and environmental changes, with the solute phase and droplet phase undergoing mutual transformation and distribution into the surrounding environment as conditions shift [3,4]. Among these, the core dynamic property lies in the multivalent interactions among biomacromolecules, which serve as the primary driving force for the phase separation. These interactions, which include protein-protein, protein-nucleic acid, and nucleic acid-nucleic acid associations, involve electrostatic interactions, hydrogen bonds, and hydrophobic interactions (Figure 2). The existence and synergy of these multivalent interactions prompt proteins and nucleic acids to form condensate structures with specific functions within cells, exhibiting dynamic changes. Notably, low-complexity domains or intrinsically disordered regions (IDRs) in proteins serve as key sites for multivalent interactions. They can multivalently recruit other molecules and effectively promote phase separation [19,20]. Environmental factors such as pH, temperature, ionic concentration, and variations in protein and nucleic acid composition allow membrane-less organelles or condensates to coexist with the surrounding cytoplasmic or nucleoplasmic environment in a dynamically regulated manner [3,21,22,23,24,25,26,27,28]. Understanding phase separation provides insights into biological processes, including gene regulation, stress response, and the pathogenesis of diseases. As research advances, manipulating phase separation holds potential for novel therapeutic strategies and biomaterial development.

### 2.2. Multivalent Interactions in Phase Separation

In biochemistry, proteins exhibit a modular architecture, with evolutionarily conserved regions that fold into distinct domains. Some of these proteins have multiple copies of the same domain with a specific topological arrangement in a molecule or complex, a phenomenon known as multivalency. Multivalent interactions, characterized by multiple weak binding forces such as electrostatic interactions, hydrogen bonds, and hydrophobic interactions, establish equilibrium and regulate various biological processes (Figure 2). These interactions allow the multivalency of individual molecules to dynamically engage with multiple partners, forming biomacromolecular condensates that transiently interact with surrounding molecules via phase separation [19,29,30,31,32,33]. p62/SQSTM1 is an autophagy receptor and signaling adaptor with an N-terminal PB1 domain. Evidence shows that the p62/SQSTM1 protein acts as a scaffold through the self-assembly of its PB1 domain, promoting the formation of autophagosomes and recruiting other autophagosome factors via additional interaction domains under various stress conditions [34,35]. Although debated, transient structures may also play a role in this process. Phase-separating molecules are classified into two categories: “scaffolds,” which are both necessary and sufficient for phase separation, and “clients”, which are recruited into condensates but are not essential for their formation. Scaffold proteins in phase separation are the driving molecules in the process of phase separation. They can self-assemble to form droplets and recruit client proteins. PodJ is a scaffold protein that self-assembles through phase separation to form a polar signaling complex in the model bacterium, Caulobacter crescentus. It recruits specific client proteins, PleC (phosphatase PleC) and DivJ (protein kinase DivJ), which play a role in determining cell polarity [36,37]. Overall, multivalent interactions are crucial drivers of phase separation, significantly influencing the formation and properties of biomacromolecular condensates and their related biological functions.

### 2.3. The Role of IDRs in Phase Separation

Emerging evidence indicates that proteins in phase-separated condensates contain IDRs or interact via interactions between folded domains and short linear motifs. IDRs, with their flexible and highly charged sequences, facilitate various weak interactions with other biomolecules, promoting phase separation [38,39,40,41,42,43,44,45,46,47]. The “sticker and spacer” framework is a model for understanding intrinsically phase-separated IDRs. In this framework, “sticker” residues participate in weak binding interactions, while “spacer” residues modulate the properties of the resulting condensates. Proteins such as Fused in Sarcoma (FUS), TAR DNA-binding protein 43 (TDP43), and Heterogeneous Nuclear Ribonucleoprotein A1 (hnRNPA1) form dynamic phase separation condensates through RNA binding. In the IDRs of these proteins, arginine (R) or tyrosine (Y) of certain amino acids act as “stickers” to participate in interchain attractive interactions, while the linking residues that connect these “stickers” serve as “spacers” [48,49].

IDRs facilitate weak interactions with RNA molecules, thereby promoting the formation of biomolecular condensates. RNA-binding proteins interact with RNA molecules via electrostatic and hydrogen bond interactions, leading to the formation of RNA-protein condensates [50]. The N-terminal IDRs of the yeast prion protein Sup35 and the Drosophila RNA-binding protein Me31b prevent adjacent folded domains from forming kinetically trapped (i.e., “irreversible”) condensates and instead promote the formation of reversible liquid-like assemblies. IDRs also serve as regulatory elements linking phase separation to environmental cues. Their conformational flexibility allows them to respond to changes in cellular conditions. The hydrophobicity of the IDR of the yeast RNA-binding protein Pab1 can precisely regulate condensate formation according to temperature changes, ensuring physiological adaptability in the natural environment. This responsiveness enables cells to rapidly adjust the formation and dissolution of biomolecular condensates in response to external stimuli. IDRs are indispensable for the formation and regulation of biomolecular condensates through phase separation. Their unique structural features and dynamic interactions provide a versatile platform for the assembly of functional cellular compartments and modulation of essential biological processes.

### 2.4. The Environment and the Occurrence of Phase Separation

Changes in the external environment, including fluctuations in temperature, pressure, and concentration, trigger phase separation. This occurs by disrupting the pre-existing interaction patterns among the components of the system, causing the different phases to segregate spontaneously. When the concentrations of biomacromolecules reach a certain threshold, the strengthened molecular interactions can overpower thermal motion, ultimately leading to phase separation. Temperature variations have a significant impact on the conformation and interactions of biological macromolecules. Some biomacromolecules are more prone to phase separation at low temperatures [51]. Conversely, under high-temperature conditions, plant ALBA (acetylation lowers binding affinity) proteins undergo reversible phase separation to form cytoplasmic granules rich in poly (A) tail mRNA, recruiting HSF mRNAs into stress granules and processing bodies to protect them from degradation and enhance plant heat tolerance [27]. Similarly, pH changes influence the occurrence of phase separation by altering the charge state of biomacromolecules, which impacts electrostatic interactions [52].

Post-translational modifications (PTMs) act as molecular rheostats that fine-tune phase separation in response to environmental stress. Under oxidative stress, the AMPK-mediated phosphorylation of prion-like domains (PLDs) in scaffold proteins like TDP-43 disrupts π–cation interactions, dissolving condensates to maintain proteostasis [53,54]. Scaffolding proteins and nucleic acids further dynamically modulate liquid-liquid phase separation thresholds: hypoxia upregulates stress granule-nucleating proteins, while osmotic stress elevates lncRNAs like NEAT1, to stabilize paraspeckles [55]. DNA damage exemplifies context-specific regulation, where PARylation-dependent phase separation of 53BP1 forms repair foci that compartmentalize chromatin remodelers to ensure genomic integrity [56]. These mechanisms converge through environmental sensors that coordinate PTMs and biomolecule availability to regulate the phase separation plasticity.

## 3. The Role of Phase Separation in Detecting and Transducing Environmental Signals

### 3.1. Phase Separation for Phenotypic Plasticity in Response to Environmental Changes

Phase separation is a crucial intracellular regulatory mechanism that exerts a significant influence on phenotypic plasticity, endowing organisms with the capacity to adapt to diverse environmental conditions. Phenotypic plasticity, defined as the ability of an organism to modify its phenotype in response to environmental alterations, is driven by the regulation of gene expression and intracellular molecular mechanisms, with phase separation playing a central role in this process.

In complex and dynamic environmental settings, organisms are compelled to continuously fine-tune their phenotypes to ensure their survival. Phase separation participates in orchestrating this plasticity by modulating the aggregation and distribution of biomacromolecules within cells, affecting cellular functions and metabolic activities. Specifically, it promotes the formation of functional condensates or membrane-less organelles that precisely regulate intracellular signal transduction, gene expression, and metabolic pathways. During development, the phase separation of cell fate determinants gives rise to distinct intracellular microenvironments that influence gene expression and the direction of cell differentiation. These microenvironments function as metabolic regulatory hubs, optimizing resource utilization and safeguarding cell survival and growth [37,57]. Furthermore, phase separation impacts intercellular interactions and tissue and organ development by regulating the distribution and activity of cell surface receptors and signaling molecules. This modulation has a profound effect on signal transmission, cell differentiation, migration, proliferation, and apoptosis, enabling organisms to fine-tune their developmental patterns and adapt more effectively to environmental changes [58,59,60].

**Figure 3:**
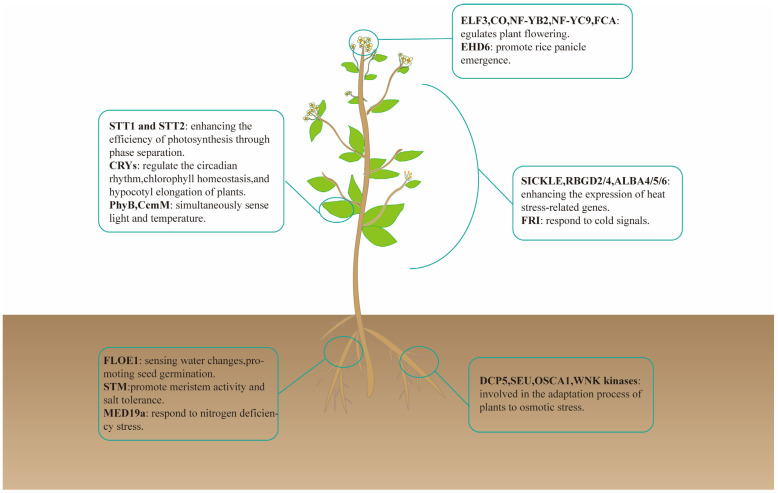
The multifaceted role of separation in plant stress adaptation. Plants can respond to environmental stresses through phase separation. This phenomenon is evident in various physiological processes, including seed germination, the regulation of flowering time, photosynthesis, and the root’s perception of nitrogen signals.

**Figure 4:**
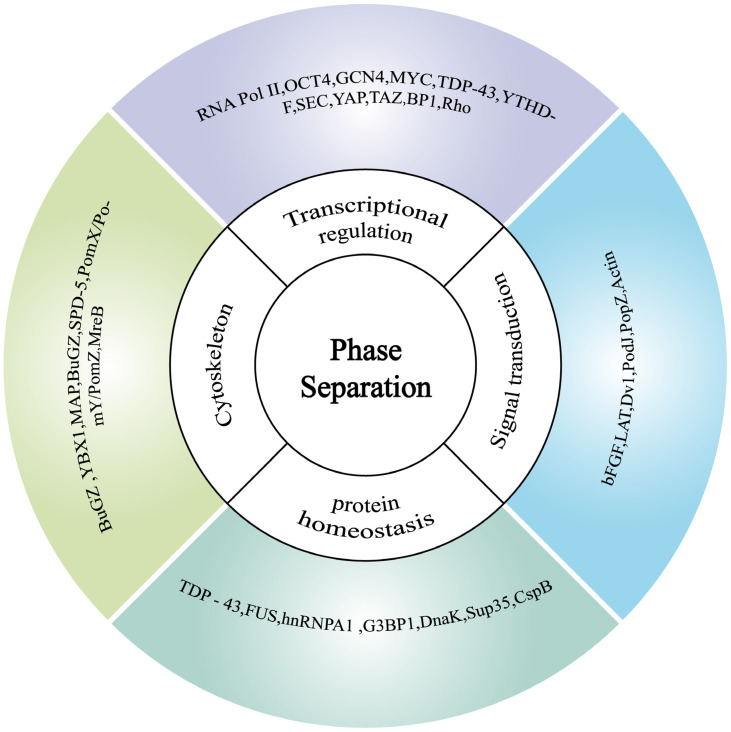
Phase separation enhances animal and microbial stress adaptation through four aspects: signal transduction, transcriptional regulation, cytoskeleton, and protein homeostasis. The genes shown in the figure are as follows.

Phase separation is involved in regulating phenotypic plasticity across diverse biological groups, including plants (Figure 3), animals, and microorganisms (Figure 4). By augmenting environmental adaptability, this mechanism ensures the survival and reproduction of organisms under diverse conditions. Overall, phase separation is a fundamental process that underlies the dynamic adaptability of life in ever-changing environments.

The genes shown in the figure are as follows: ELF3 (E74-like ETS transcription factor 3) [61], CO (CONSTANS) [62], NF-YB2 (Nuclear Factor Y subunit B2) and NF-YC 9 (Nuclear Factor Y subunit C9) [63], FCA (FLOWERING CONTROL LOCUS A) [64], EHD6 (EARLY HEADING DATE 6) [65], STT1 (Stromal Tetratricopeptide Repeat Protein 1) and STT2 (Stromal Tetratricopeptide Repeat Protein 2) [66], CRYs (Cryptochromes) [67], PhyB (Phytochrome B [68]), CcmM (Carboxysome Component M [69]), SICKLE [70], RBGD2 (RNA-binding glycine-rich group D 2), RBGD4 (RNA-binding glycine-rich group D 4) [71], ALBA4 (AT1G20220), ALBA5 (AT1G76010), and ALBA6 (AT3G07030) [27], FRI (FRIGIDA) [72], FLOE1 (At4g28300) [6], STM (SHOOT MERISTEMLESS) [73], MED19a (Mediator Complex Subunit 19a) [74], DCP5 (DECAPPING 5) [75], SEU (SEUSS) [26], OSCA1 (Hyperosmolality-induced) [76,77], WNK kinases (With No Lysine (K) Kinases) [78], and EHD6 (EARLY HEADING DATE 6) [65].

#### 3.1.1. Phase Separation in Plants: Regulating Phenotypic Plasticity in Response to Environmental Variations

In plant life, phase separation has emerged as a crucial regulatory mechanism that finely tunes phenotypic plasticity to adapt to ever-changing environmental conditions. Membrane-less organelles formed by phase separation within plant cells exhibit remarkable capacity to dynamically respond to environmental signals. This dynamic responsiveness is pivotal in adjusting the growth, development, and physiological status of plants. For example, stress granules (SGs), a type of phase-separated structure, are formed in response to various environmental stresses, including extreme temperature variations and drought conditions. During these challenging circumstances, RNA-binding proteins and mRNA undergo phase separation to form SGs that serve as temporary storage units for mRNA, effectively safeguarding it against mistranslation or degradation. Once stress is alleviated, protein synthesis promptly resumes, ensuring the seamless maintenance of normal cellular functions [79,80].

RNA Pol II (RNA Polymerase II) [81]; GCN4 (General Control Nonderepressible 4) and OCT4 (Octamer-binding transcription factor 4) [82]; MYC (Myc proto-oncogene protein) [83]; TDP-43 (TAR DNA-binding protein 43) [84]; YTHDF (YTH domain family protein) [85]; SEC (super elongation complex) [86]; TAZ (Transcriptional Coactivator with PDZ-binding Motif) and YAP (Yes1 Associated Transcriptional Regulator) [87]; BP1 (Bromo-adjacent homology-plant homeodomain domain containing protein 1) [88]; Rho (Rho guanosine triphosphatase) [89]; BuGZ [90]; YBX1 (Y-box binding protein 1) [91]; MAP (Mitogen-Activated Protein Kinase) [92]; SPD-5 (pindle-defective protein 5) [93]; PomX/PomY/PomZ (Pole-organizing protein X; Pole-organizing protein Y; Pole-organizing protein Z) [94]; MreB (Cell shape-determining protein MreB) [95]; bFGF (Basic Fibroblast Growth Factor) [96]; LAT (Linker for Activation of T-cells) [59]; Dv1 (Disheveled protein 1) [28]; PodZ (Pole-organizing protein Z) [37]; Actin [97]; FUS (Fused in Sarcoma) [98]; hnRNPA1 (Heterogeneous nuclear ribonucleoprotein A1) [99]; G3BP1 (Ras-GTPase-activating protein (GAP)) [100]; DnaK (DnaK chaperone protein) [101]; Sup35 [12]; CspB (Cold shock protein B) [102].

Moreover, plant hormone signal transduction is closely associated with phase separation. Auxin, a hormone of paramount importance in the context of plant growth and development, has specific proteins within its signal transduction pathway that have the unique ability to form droplet-like signal transduction complexes via phase separation. These complexes tend to localize within specific regions of the cell, significantly enhancing the efficiency and specificity of signal transduction. The auxin receptor TIR1 and its co-receptor, ABP1, interact through phase separation to form a functional signal transduction center that regulates plant growth direction and organ development. This sophisticated mechanism empowers plants to adjust their morphology in response to environmental factors, such as light and gravity [103].

#### 3.1.2. Phase Separation in Animals: Regulating Phenotypic Plasticity to Adapt to Environmental Changes

Phase separation is crucial for regulating phenotypic plasticity in animals, enabling adaptation to environmental changes through various biological processes. Synaptic plasticity in neuronal cells is an essential process for behaviors such as learning and memory, which are regulated by phase separation. The postsynaptic density (PSD), which is rich in proteins and signaling molecules, forms functional regions through phase separation [104,105,106,107]. These regions dynamically regulate synaptic signal transmission and integration. When animals are exposed to environmental stimuli, phase separation at the synapses adjusts synaptic plasticity for adaptive responses.

From the perspectives of individual development and physiological adaptation, phase separation plays a significant role (Figure 4). It influences cellular organization and function, enabling precise control of developmental processes and adaptive responses. Phase separation is crucial for both development and adaptation by organizing biomolecules into distinct compartments. In Drosophila melanogaster embryonic development, transcription factors form condensates via phase separation to regulate gene expression. In terms of physiological adaptation, phase separation is involved in processes like immune responses and metabolic regulation. It influences immune signal transduction and the activation of immune cells. The key protein STING (Stimulator of Interferon Genes) in the innate immune signaling pathway of animals forms endoplasmic reticulum cubic membrane structures through phase separation. These structures are referred to as “STING Phase Separators”. These structures negatively regulate the cGAS-STING pathway, preventing overactivation of innate immunity [11]. In adipocytes, phase separation modulates lipid metabolism-related proteins, aiding animals in adapting to variable conditions.

#### 3.1.3. Phase Separation in Microorganisms: Regulating Phenotypic Plasticity to Adapt to Environmental Fluctuations

In bacteria, phase separation is crucial for physiological processes. When faced with environmental stresses such as nutrient deficiency, osmotic pressure changes, or antibiotic stress, bacteria form intracellular structures through phase separation. Upon environmental improvement, bacterial condensate dissolution triggers cellular rejuvenation and growth resumption. The RNA chaperone proteins of *Escherichia coli* stabilize mRNA and regulate translation priority during nutritional deficiency. Phase separation of the RNA-binding protein Hfq mediates stress-responsive RNA condensation and polar aggregation via its RNA-binding domain (which is necessary for the process) and C-terminal IDRs in a TmaR-dependent manner [108]. Stress response proteins create condensates that protect DNA, RNA, and proteins, optimize resource use, and support survival (Figure 4).

In fungi, such as yeast, phase separation is key to phenotypic plasticity. Yeast cells adapt their morphology and physiology to environmental conditions. Proteins form subcellular structures through phase separation, which affects growth, metabolism, and stress response. Under nutrient-deficient conditions, autophagy-related proteins undergo phase separation to form pre-autophagosomal structures, recycling intracellular substances and aiding survival [109]. Moreover, phase separation regulates yeast reproduction and enables yeasts to adjust their reproductive mode in response to nutritional status and spatial conditions in the environment, ultimately improving their survival adaptability [12,110].

Phase separation also regulates the collective behavior of microorganisms. During biofilm formation, bacteria secrete extracellular polysaccharides, proteins, and other substances to form protective three-dimensional structures. This strategy shields them from antibiotics and immune response. Phase separation aids in the formation of biofilms by creating condensates that act as a framework, promoting stability and resistance to external threats.

### 3.2. The Regulation of Stress Responses by Phase Separation

**Figure 5:**
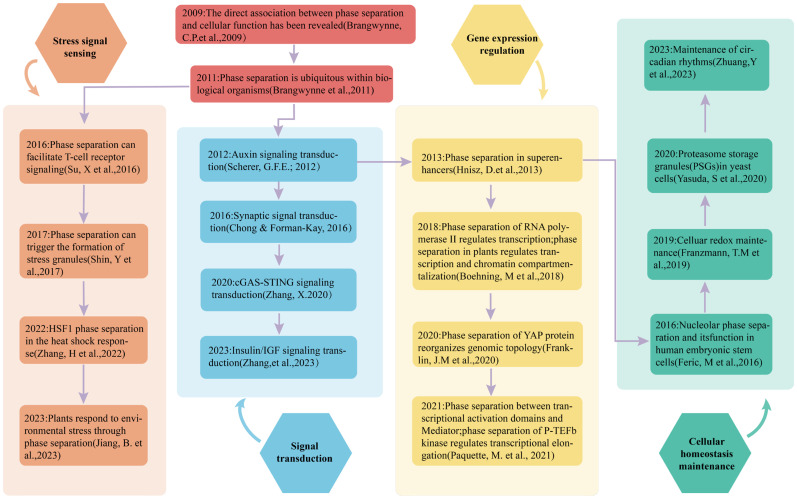
Significant outcomes in phase separation research and categorized into four distinct domains: signal sensing, signal transduction, gene expression regulation, and cellular homeostasis maintenance. Within each domain, the major milestone research findings are presented in chronological order [4,15,16,28,40,50,53,59,80,87,90,103,111,112,113,114,115,116].

When organisms face internal and external environmental stimuli, they initiate adaptive and defensive responses to maintain internal environmental homeostasis and ensure survival, which are defined as stress responses. Extreme environments are those beyond an organism’s normal tolerance range, while quasi-extreme environments occur locally due to specific physiological or pathological processes. Phase separation plays a crucial regulatory function in these stress responses by enabling organisms to sense stress signals, regulate signal transduction pathways, and modulate gene expression, thus facilitating adaptation and survival (Figure 5).

#### 3.2.1. Phase Separation in Stress Signal Perception

Organisms need to be responsive to environmental signals to initiate adaptive responses. Phase separation plays a crucial role in sensing these signals, as biomolecular condensates directly perceive alterations in physical and chemical environmental cues [111,117,118]. For temperature fluctuations, it aids adaptation by forming stable membrane regions at low temperatures to enhance the resistance. At high temperatures, heat shock proteins promote phase separation to protect membrane stability. In plants, ELF3 utilizes phase separation to sense temperature changes and recruit proteasomes into SGs to maintain protein homeostasis [61,119]. Phase separation protects biomacromolecules from humidity and osmotic pressure changes. In hyperosmotic environments, proteins aggregate to maintain a water balance. The Decapping 5 (DCP5) protein in *Arabidopsis* thaliana rapidly condenses under high osmotic stress, enhancing the plant’s perception of osmotic stress through phase separation [75]. Phase separation influences membrane permeability, forms membrane-less organelles for substance regulation, and maintains cell metabolism. When osmotic pressure varies, phase separation facilitates condensate formation, reducing macromolecule exposure to adverse conditions, regulating their concentration and distribution, stabilizing membrane structure, and participating in cellular quality control [26,75].

#### 3.2.2. Phase Separation in Signal Transduction

Receptor proteins may undergo conformational changes and form condensates upon detecting environmental signals. These condensates recruit and activate downstream signaling molecules, thereby amplifying and propagating the signal. In the Wnt signaling pathway, protein–ligand binding forms condensates that activate downstream transcriptional programs. Phase separation also isolates and protects signaling molecules from degradation or inactivation, allowing their rapid release and activation when external conditions change [120,121]. Condensates gather dispersed proteins, increasing the interaction probability and improving signal transduction efficiency [122]. The dynamic nature of phase separation allows rapid regulation of signal responses [123,124]. During the inflammatory stress response, proteins in the NF-κB signaling pathway aggregate through phase separation to form a signal transduction center [125,126].

#### 3.2.3. Phase Separation in Stress Response Gene Expression

When cells encounter various stress stimuli, phase separation finely regulates gene expression through multiple mechanisms to facilitate adaptation. At the transcriptional level, phase separation primarily regulates the expression of stress response genes by modulating the activity and localization of transcription factors and affecting chromatin accessibility. Meanwhile, phase separation alters chromatin structure and accessibility, which are crucial for gene transcription. These condensates interact with chromatin, loosening or compacting specific regions, thereby affecting the binding of transcription factors and regulatory factors to DNA. Under stress, transcription factors and co-regulators form high-concentration condensates that act as platforms to recruit RNA polymerase and related proteins, thus promoting stress response gene transcription. During heat shock, heat shock transcription factors (HSFs) form condensates that bind to heat shock protein gene promoters, activating their transcription and supporting protein homeostasis [112,127].

At the post-transcriptional level, phase separation influences mRNA stability and translation efficiency. RNA-binding proteins form condensates that selectively bind stress response-related mRNAs. This binding protects mRNAs from degradation and promotes translation to ensure rapid synthesis [128,129]. During oxidative stress, chromatin remodeling proteins undergo phase separation to enable the efficient expression of antioxidant stress-related genes. Phase separation plays a multifaceted role in regulating stress response gene expression, from transcriptional initiation to post-transcriptional regulation, thereby supporting cell survival and adaptation under stress.

### 3.3. Phase Separation in Cellular Homeostasis During Environmental Variation

Phase separation is crucial for cellular homeostasis, enabling the compartmentalization and regulation of biochemical reactions in the absence of membrane-bound organelles. This dynamic organization allows cells to swiftly adapt to changes, ensuring precise control of processes such as protein synthesis, degradation, and stress responses. By modulating biomolecule interactions, phase separation stabilizes the internal environment and preserves cellular function amid external fluctuations. It aids adaptation by forming condensates with stalled translation initiation complexes, RNA-binding proteins, and non-translated mRNAs, isolating and protecting them for rapid translation resumption after stress [130,131,132]. Enhanced proteasome activity within these condensates also aids in disassembly, improving thermotolerance and maintaining homeostasis [119]. Phase separation is crucial for the assembly and dynamics of condensates, which are driven by weak multivalent interactions between RNA and proteins [132]. The composition and properties of condensates change with the type and duration of stress, highlighting the plasticity of the stress response [133]. Photocatalytic proximity labeling technology has revealed dynamic changes in the transcriptome of condensates, analyzing core component variations under unstimulated conditions and the recovery phase, thus expanding our understanding of cytoplasm-based membrane-less organelles formed by phase separation [132].

#### 3.3.1. Phase Separation in Constructing Intracellular Functional Zones for Cellular Homeostasis

Intracellular homeostasis is essential for normal physiological functions, and phase separation is involved in maintaining various cellular homeostatic states. By forming membrane-less organelles, phase separation constructs functional zones within the cell. Specific molecules, such as P granules, aggregate to create these organelles, establishing a specific cellular order and providing insights into their formation. These membrane-less organelles contribute to cell development and function maintenance at different stages [15]. In chloroplasts, phase separation is crucial for functional homeostasis. The key protein transport and sorting factors, Stt1 and Stt2, form condensed droplets through phase separation, facilitating the movement of substrates from the chloroplast stroma to the thylakoid membranes. This process ensures the normal operation of chloroplasts and is crucial for maintaining homeostasis in physiological processes [66]. Additionally, phase separation maintains the cytoskeletal structure and function. The end-binding protein EB1, through its interaction with microtubule ends, regulates the microtubule plasticity of the cell division spindle, forming dynamic aggregates that stabilize microtubules and control their growth direction, thus ensuring proper cytoskeletal maintenance [134].

#### 3.3.2. The Role of Phase Separation in Protein Homeostasis

Phase separation regulates protein homeostasis by organizing protein interactions and facilitating their degradation to maintain cellular stability and function. The SERRATE protein, a plant-specific C2H2 zinc-finger protein regulatory factor, forms condensates via phase separation and liquid co-condensates with MTB protein. This interaction prevents the MTB from forming insoluble aggregates, protects proteins from 20S proteasome degradation, and maintains protein homeostasis [135,136]. Cells dynamically regulate protein degradation activity through ubiquitin-dependent proteasome phase separation and rapidly respond to the imbalance of protein homeostasis caused by stress [113]. This finding reveals the key role of phase separation in regulating protein degradation, providing a new perspective on the mechanism of protein homeostasis.

#### 3.3.3. Phase Separation in Gene Transcription for Cellular Homeostasis

Phase separation influences transcription factor dynamics and chromatin organization by compartmentalizing transcription factors and coactivators into these condensates. This affects transcription machinery assembly at genomic loci and regulates gene expression. For example, the MYC gene forms condensates at super-enhancer regions, which is related to its high-level expression associated with cell proliferation and tumorigenesis [83,137]. Similarly, BRD4 binds to super-enhancers to promote the expression of inflammation- and cancer-related genes [138,139]. Super-enhancers form condensates through phase separation, enrich transcription factors, and promote gene transcription, which is essential for cell identity and function. Abnormal super-enhancer phase separation, such as that involving p53, leads to cellular dysfunction and disrupts intracellular homeostasis [114]. The lncRNA, MTAR1, is also critical for the post-transcriptional regulation of MYC-induced tumorigenesis. MTAR1 recruits IGF2BPs into PABP1-mediated phase separation complexes and facilitates post-transcriptional regulation [140].

In the cell nucleus, the phase separation of chromatin-associated proteins is crucial for maintaining chromatin structure integrity and regulating gene expression. Cohesin and other chromatin-associated proteins utilize phase separation to form chromatin loops, which indirectly impact chromatin structure and gene expression [141]. Phase separation enables biomolecule aggregation and the creation of functional zones that maintain nucleolar structural and functional homeostasis. The METTL3/METTL14 complex aids in degrading SUV39H1/H2, preventing the buildup of H3K9me3, and ensuring proper phase separation, which supports rRNA synthesis and ribosomal assembly, both of which are crucial for cell homeostasis [142]. Under physiological conditions, phase separation regulates protein quality by preventing misfolding and aggregation. However, these abnormalities lead to pathological aggregates that disrupt intracellular homeostasis [143].

## 4. Phase Separation in Biological Adaptation and Evolution

Phase separation is integral to biological adaptation, significantly impacting evolution and biodiversity (Table 1). It is involved in physiological processes, such as photosynthesis and stress responses [26,144,145,146,147]. Phase separation provides a dynamic platform for the spatiotemporal organization of biomolecules, driven by multivalent interactions between the intrinsically disordered regions of proteins and nucleic acids [4], enabling cells to rapidly redistribute their components in fluctuating environments—a molecular strategy that effectively bridges short-term adaptation and long-term evolutionary innovation.

In plants, phase separation is a flexible and efficient regulatory mechanism that allows plants to adapt to complex and changing environments [27,152,153,154]. Field studies on *Arabidopsis thaliana* have revealed that drought-induced nuclear speckles, enriched with RNA-binding proteins FCA and FPA, orchestrate stress-responsive gene splicing through phase separation-mediated compartmentalization [64]. Parallel investigations have demonstrated chloroplast adaptability: the C-terminal IDR of Rubisco activase undergoes light-dependent phase transitions to maximize photosynthetic output under variable irradiance [69]. By dynamically regulating gene expression and protein activity, phase separation enables plants to thrive in diverse ecological niches, contributing to their evolutionary success and maintenance of biodiversity.

In animals, phase separation is pivotal in crucial cellular processes. In addition to stress granule formation, phase separation governs developmental processes such as embryogenesis. In *Drosophila* oocytes, the Balbiani body, a phase-separated condensate enriched with mitochondria and mRNAs, ensures the asymmetric distribution of maternal determinants, which are critical for embryonic patterning [93,155]. During viral infection or oxidative stress, RNA-binding proteins form stress granules through phase separation, temporarily storing mRNA and proteins to prevent their degradation. Once the stress subsides, mRNA is released for translation, aiding in the restoration of cellular function [155,156]. However, this adaptive mechanism has an evolutionary trade-off. Clinical data associate persistent stress granule formation with neurodegenerative pathologies like ALS, suggesting that selective pressures balancing cellular protection and proteostatic risks have shaped phase separation regulation in metazoans [79]. In microorganisms, phase separation creates membrane-less organelles with specialized functions. In extremophiles like *Sulfolobus acidocaldarius*, the phase separation-mediated assembly of heat-shock proteins enhances thermotolerance by sequestering denatured proteins [12]. These condensates enhance biochemical efficiency and environmental adaptability, thereby offering evolutionary advantages. By enabling biomolecules to recombine into functional modules, phase separation facilitates precise responses to stimuli and promotes phenotypic plasticity, driving microbial diversification in terms of morphology, physiology, and ecology [157,158].

From an evolutionary perspective, phase separation is a conserved and versatile mechanism. Comparative studies have shown that IDRs are evolutionarily ancient and enriched in taxon-specific proteins, suggesting the co-option of phase separation for lineage-specific adaptations [159]. For example, fungal prion domains evolved from disordered regions to generate heritable phenotypic diversity, enabling rapid adaptation to environmental challenges [110].

Over time, this mechanism has optimized regulatory networks, promoting microbial diversity in terms of morphology, physiology, and ecology, thereby providing microorganisms with a competitive advantage. It regulates gene expression to adapt to various conditions, driving evolutionary branching and enriching biodiversity, and thus becoming a crucial force in microbial evolution [3]. Thus, phase separation functions as both an immediate stress response mechanism and a driver of long-term evolutionary innovation through phenotypic diversification. Its integration with genetic and epigenetic networks underscores its central role in the emergence of biological complexity [160].

## 5. Conclusions

Phase separation is an important biological phenomenon that plays an indispensable role in the adaptation of organisms to complex environments. It aids survival and reproduction by perceiving signals, regulating phenotypic plasticity, controlling stress responses, and maintaining cellular homeostasis. Despite the progress made in phase separation studies, critical challenges remain.

Most studies rely on reductionist in vitro systems that poorly replicate cellular crowding conditions. Recent studies have shown that condensate properties differ markedly between purified proteins and native cellular environments, e.g., nucleolar phase separation requires chromatin as a scaffold in vivo but forms spontaneously in vitro [116]. Real-time tracking of condensate maturation (e.g., liquid-to-solid transitions in neurodegenerative aggregates) remains technically challenging [161].

How do different signaling cascades integrate with key proteins to trigger phase separation in a spatiotemporally controlled manner? However, the interplay between phase separation and canonical pathways, such as phosphorylation cascades, remains unclear. Although stress granules coordinate with the ubiquitin-proteasome system to control protein quality [162], the mechanism by which phase separation prioritizes signaling in competing pathways during simultaneous stress (e.g., heat + oxidative stress) remains unknown.

Advances in high-resolution imaging (e.g., MINFLUX nanoscopy [163]), computational methods (AI-driven phase-field modeling [164]), and single-molecule techniques (DNA-PAINT for condensate stoichiometry [165]) will help address these gaps [166,167]. Integrating phase separation studies with systems biology approaches [168]—such as constructing whole-cell LLPS interaction maps—could reveal hierarchical regulatory principles [129].

Phase separation is multifunctional across biological contexts, from bacteria to higher eukaryotes, and has significant implications for evolution. Future research should adopt a multidisciplinary approach to explore the origins and evolution of phase separation mechanisms, their role in environmental adaptation, and the evolutionary history of related genes and regulatory elements. Researchers are now leveraging phase separation mechanisms to engineer adaptive biomaterials, rewire cellular metabolism, and develop targeted therapies for conditions like Alzheimer’s disease, while simultaneously engineering crop variants with enhanced drought tolerance through controlled biomolecular condensation. A comprehensive understanding of these applications will offer new insights into molecular adaptation mechanisms, benefiting the fields of biomedicine, biotechnology, human health, and environmental protection.

## Figures and Tables

**Table 1:** Phase separation mediates stress responses to enhance an organism’s environmental adaptability.

Type	Protein Name	Features Associated with Phase-Separation Capacity	Nature of Stress	References
Plants	OsCRT3, OsCIPK7	Cold stress triggers secondary structural changes in OsCRT3 and enhances its binding affinity with OsCIPK7, which finally boosts its kinase activity.	Cold	[148]
Plants	TANDEM ZINC-FINGER/PLUS3 (TZP)	TZP’s phase separation promotes PPKs and phyA colocalization and interaction, enhancing PPK-mediated phyA phosphorylation in FR light, crucial for plant photomorphogenic development in FR-rich conditions like canopy shade.	Light	[118]
Plants	CRY2/SPA1/FIO1	The blue light receptor cryptochrome 2 (CRY2) and the METTL16-type m6A writer FIONA1 (FIO1) regulate chlorophyll homeostasis in response to blue light.	Light	[111]
Plants	ELF3	The ability of temperature to rapidly shift ELF3 between active and inactive states via phase transition represents a previously unknown thermosensory mechanism.	Heat	[61]
Plants	RALF	RALF-pectin phase separation mediates an exoskeletal mechanism to broadly activate FER-LLG1-dependent cell surface responses to mediate the global role of FER in plant growth and survival.	Salt, heat	[149]
Plants	PhyB	The N-terminal extension’s disorder and C-terminal structure’s oligomerization drive phase separation, with the NTE sensing temperature signals.	Temperature light	[144]
Plants	SEU	The condensation of SEU is essential for plant tolerance to osmotic stress.	Osmotic pressure	[2]
Plants	RBGD2/4	Tyrosine enrichment in the low-complexity domain (LCD) drives phase separation, recruiting heat-tolerant proteins and transcripts to stress granules (SGs).	Heat	[3]
Plants	NPR1	Conserved cysteine clusters in IDRs mediate phase separation to form SINCs, enriching proteins that regulate cell death and stress responses, thus promoting cell survival under stress.	Salt	[4]
Plants	ALBA4/5/6	Heat stress induces ALBA’s phase separation, aiding HSF mRNA recruitment to SGs and P-bodies, inhibiting HSF mRNA degradation.	Heat	[5]
Plants	FRI	Low-temperature signals induce FRI nuclear condensates, dissociating FRI from the FLC gene. COOLAIR RNA promotes FRI condensate accumulation, stabilizing FRI proteins for rapid temperature response.	Temperature	[6]
Plants	STM	Under salt stress, it enhances transcriptional regulation, promoting meristematic activity and salt tolerance.	Salt	[7]
Plants	FLOE1	FLOE1 mediates the response to water stress and regulates seed germination.	Water	[8]
Animal/microorganisms	RNA polymerase (Pol) II	Pol II forms clusters or hubs at active genes through interactions between CTDs and activators, and CTD phosphorylation liberates Pol II enzymes from hubs for promoter escape and transcription elongation.	Heat	[115]
Animals	TIFA	As a sensor of upstream signals, the phase separation process of TIFA leads to the formation of membrane-less condensates within the ALPK1-TIFA-TRAF6 pathway, providing a potential application direction for the development of therapeutic biotechnology.	Disease	[10]
Animals	WNK kinases	WNK kinases are physiological crowding sensors that phase separate to coordinate a cell volume rescue response.	Hyperosmotic stress-induced	[11]
Animals	FUS	FUS is enriched in the nucleus and involved in transcription, DNA repair, and RNA biogenesis	Disease	[98]
Animals	Glycyl-tRNA synthetase (GlyRS)	GlyRS is translocated from the cytoplasm into SGs upon stress, where the mutant GlyRS perturbs the G3BP-centric SG network by aberrantly binding to G3BP. This disrupts SG-mediated stress responses, leading to increased stress vulnerability in motoneurons. Disrupting this aberrant interaction rescues SG abnormalities and alleviates motor deficits in CMT2D mice.	Peripheral neuropathy	[13]
Animals	MeCP2	MeCP2 enhances the separation of heterochromatin and euchromatin through its condensate partitioning properties, and the disruption of condensates may be a common consequence of mutations in MeCP2 that cause Rett syndrome.	Disease	[14]
Microorganisms	53BP1	This liquid droplet-like behavior of 53BP1 compartments might help to coordinate local lesion recognition with global gene activation in response to DNA damage.	Ionizing radiation	[150]
Microorganisms	Ded1p	Ded1p condensation inactivates Ded1p, represses housekeeping mRNA translation, and promotes stress mRNA translation. It is adaptive and fine-tuned to the organism’s maximum growth temperature, and part of an extended heat shock response that selectively inhibits housekeeping mRNA translation to enhance survival in severe heat stress.	Heat	[17,151]
